# Determination of plasma and leukocyte vitamin C concentrations in a randomized, double-blind, placebo-controlled trial with Ester-C^®^

**DOI:** 10.1186/s40064-016-2605-7

**Published:** 2016-07-25

**Authors:** Susan H. Mitmesser, Qian Ye, Mal Evans, Maile Combs

**Affiliations:** 1 Department of Nutrition & Scientific Affairs, NBTY, Inc., Ronkonkoma, NY 11779 USA; 2 KGK Synergize, Suite 1440, One London Place 255 Queens Avenue, London, ON N6A 5R8 Canada

**Keywords:** Ascorbic acid/vitamin C bioavailability, Ester-C^®^, Leukocytes

## Abstract

**Background:**

Rapid uptake of vitamin C into blood and retention in tissues are important indicators of the efficacy of vitamin C supplementation and its immune-supporting role. The objective of this study was to evaluate the bioavailability of vitamin C in plasma (reflective of recent intake) and leukocytes (reflective of tissue stores and influences on immune function) from a novel vitamin C formulation, Ester-C^®^.

**Methods:**

The study was a double-blind, placebo-controlled, crossover trial. Thirty-six subjects, 18–60 years of age, were randomized to receive placebo (PL, 0 mg vitamin C), ascorbic acid (AA, 1000 mg vitamin C), and Ester-C^®^ (EC, 1000 mg vitamin C). Plasma and leukocyte vitamin C were measured baseline and at 2, 4, 8 and 24 h postdose.

**Results:**

The concentration and percent change from baseline in plasma were significantly higher with EC at all time points when compared to PL. No significant differences between EC and AA were observed in plasma concentration. Maximum plasma concentration was higher for EC compared to AA (P = 0.039) and PL (P < 0.001). Plasma area under the curve (AUC_0–24h_) was higher for EC (P < 0.001) compared to PL. The concentration change from baseline in leukocyte vitamin C was increased with EC at 24 h post-dose (P = 0.036) while no significant within-group changes were observed in AA or PL at any time point. The percent change in leukocyte vitamin C concentration was higher for EC at 8 and 24 h compared to AA (P = 0.028 and P = 0.034, respectively) and PL (P = 0.042 and P = 0.036, respectively).

**Conclusions:**

A single dose of EC resulted in favorable percent change in leukocyte vitamin C concentration compared to AA and PL, indicating EC is retained longer within leukocytes.

*Trial registration* ClinicalTrials.gov Identifier NCT01852903

## Background

Vitamin C is a water-soluble vitamin essential for human health. It provides antioxidant protection to plasma lipids (American Society of Health-System Pharmacists 1998; Hemila 1997; Van Straten and Josling 2002; Biesalski et al. 2002) and is necessary for immune function (including leukocyte phagocytosis and chemotaxis), suppression of virus replication, and production of interferon (Hemila 1997). It has been suggested that a major role of vitamin C in immunity is protecting immune cells against oxidative stress generated during infections (Hemila 1997). To function as an effective antioxidant, vitamin C must be retained in the body at relatively high levels. While many mammals can produce vitamin C (Kasa 1983; Institute of Medicine 2000), humans do not have gulonolactone oxidase, which is the enzyme required for vitamin C biosynthesis, and therefore must obtain vitamin C from the diet (Nishikimi et al. 1994). Though normally found rich in fresh fruits and vegetables, amounts consumed may be lower than desired due to insufficient intakes and the lability of vitamin C with cooking and storage (Levine et al. 1999; Schleicher et al. 2009). Furthermore, environmental stressors, such as alcohol consumption and smoking, also require higher vitamin C consumption (Schectman 1993; Gueguen et al. 2003).

Consumption of dietary vitamin C supplements is an efficient way to increase vitamin C levels in the body when food sources do not meet needs. Previous studies have demonstrated that single gram-dose supplements produce transient peak plasma concentrations that are 2–3 fold higher than what is achieved through consuming 200–300 mg vitamin C throughout the day from food; in either case, plasma vitamin C concentrations typically return to steady state within 24 h after intake (Padayatty et al. 2004). Plasma vitamin C levels reflect the amount absorbed acutely from the digestive tract, but the efficacy of vitamin C largely hinges on whether it is absorbed and retained in cells and tissues, such as leukocytes. Both plasma and leukocyte vitamin C concentrations have been found to correlate with dietary intake of vitamin C (Jacob 1990); however, leukocyte vitamin C concentration is considered to be a more accurate indicator of tissue vitamin C concentrations (Lee et al. 1982). Leukocytes are able to maintain vitamin C concentrations several times higher than plasma vitamin C and leukocytes may better reflect long-term dietary intakes of vitamin C (Bergsten et al. 1990; Evans et al. 1982). Accurately assessing vitamin C levels in leukocytes is important not only for determining tissue stores, but also because leukocytes are vital in acute and chronic disease prevention and treatment. Phagocytes consume extracellular vitamin C when activated during an immune challenge, causing plasma and leukocyte vitamin C concentrations to decline rapidly during an infection and/or stress (Stankova et al. 1975). Furthermore, diseased populations, including patients with coronary artery disease (Wintergerst et al. 2006), type 1 (Ramirez and Flowers 1980) and type 2 (Akkus et al. 1996) diabetes mellitus, and asthma (Shidfar et al. 2005), have been reported to have reduced leukocyte vitamin C concentrations compared to healthy populations. Therefore, both plasma and leukocyte vitamin C concentrations are important when evaluating the bioavailability of vitamin C.

Ester-C^®^ (EC, NBTY, Inc., Ronkonkoma, NY, USA) is a form of vitamin C that contains calcium ascorbate and vitamin C metabolites, including dehydroascorbate, calcium threonate, and 4-hydroxy-5-methyl-3(2H)-furanone. Compared to ascorbic acid (AA), the most common form of vitamin C, EC can be consumed by a wider range of individuals due to its neutralized pH which allows for supplementation at high doses with greater tolerability for people who are sensitive to acidic foods (Gruenwald et al. 2006; Ye et al. 2015). Evidence suggests that EC may also have a higher bioavailability. Studies show that pre-incubation of cells with threonate results in a stimulated uptake of vitamin C in a dose-dependent manner (Fay et al. 1994; Fay and Verlangieri 1991). In rodents, higher plasma concentrations and less rapid excretion (Bush and Verlangieri 1987) as well as more available vitamin C activities (Verlangieri et al. 1991) have been observed in comparison with AA. Human studies comparing vitamin C levels of EC and AA are not consistent in plasma (Moyad et al. 2008; Wright et al. 1990; Johnston and Luo 1994; Pancorbo et al. 2008), but significantly higher leukocyte vitamin C levels have been constantly observed with EC than AA in male subjects (unpublished observations) (Moyad et al. 2008; Wright and Kirk 1990).

To confirm and expand upon the results from previous clinical trials (unpublished observations) (Moyad et al. 2008), the present study assessed the bioavailability and retention of vitamin C in plasma and leukocytes following oral administration of AA, EC or placebo (PL) in male and female subjects. We hypothesize that vitamin C from EC is better retained in plasma and leukocytes compared to AA or PL.

## Methods

### Subjects

Forty subjects (20 male, 20 female) between 18 and 60 years of age were enrolled in the study. The inclusion criteria comprised healthy subjects with a body mass index (BMI) of 18–30 kg/m^2^ who were non-smokers or who had stopped smoking for more than a year. Subjects were excluded from the study if they were pregnant, breastfeeding, or planning to become pregnant during the course of the trial; had used prescription or over-the-counter products known to interact with vitamin C within 72 h of randomization and during the trial, such as aspirin, nonsteroidal anti-inflammatory drugs (NSAIDs), aluminum containing antacids, and iron; had used proton pump inhibitors; had used multivitamins or other dietary supplements containing vitamin C within 7 days of randomization and during the trial; had gastroesophageal reflux disease within the past 3 months; had a history of significant gastrointestinal disease or a history of malabsorption, or any other condition which, in the Investigator’s opinion, may have adversely affected the subject’s ability to complete the study or its measures or which posed significant risk to the subject. Additionally, subjects agreed to consume a low-vitamin C diet during the study.

### Interventions

The investigational interventions (AA, EC, and PL) were stored at the study site in a locked, limited-access area at room temperature and were protected from light until administration. For EC and AA, a single daily dose of vitamin C was 1000 mg (two tablets, each tablet contained 500 mg vitamin C). PL tablets contained microcrystalline cellulose.

### Study design

**Fig. 1:**
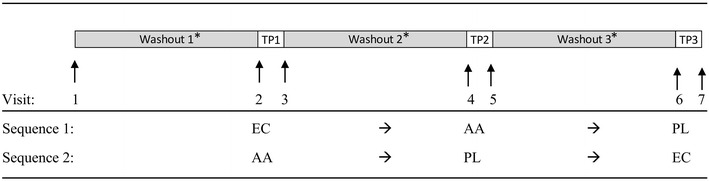
Study flow diagram. *Washout periods were > 7 days; *TP* test period (24 h), *EC* Ester-C^®^, *AA* ascorbic acid, *PL* placebo

This study was a randomized, double-blind, placebo-controlled, crossover, 24-h bioavailability study conducted at a single site, KGK Synergize Inc., London, Ontario, Canada. The study consisted of three 24-h test periods, each preceded by a 7-day washout period (Moyad et al. 2008) (Fig. 1). During the Screening Visit (Visit 1), subjects’ medical history and concomitant therapies were reviewed and eligibility was determined based on the inclusion and exclusion criteria. Height, weight, heart rate, and blood pressure were measured and BMI was calculated. For measures of general health status, blood samples were collected for hematology and clinical chemistry analysis.

This study was reviewed and approved by the Natural Health Products Directorate (NHPD), Health Canada, Ottawa, Ontario and Institutional Review Board Services, Aurora, Ontario, Canada, and was conducted in accordance with the Guideline for Good Clinical Practice (International Conference on Harmonization [ICH]) and ethical principles according to The Declaration of Helsinki. Written informed consent was obtained from each subject. The trial was registered with ClinicalTrials.gov (Identifier NCT01852903).

**Fig. 2:**
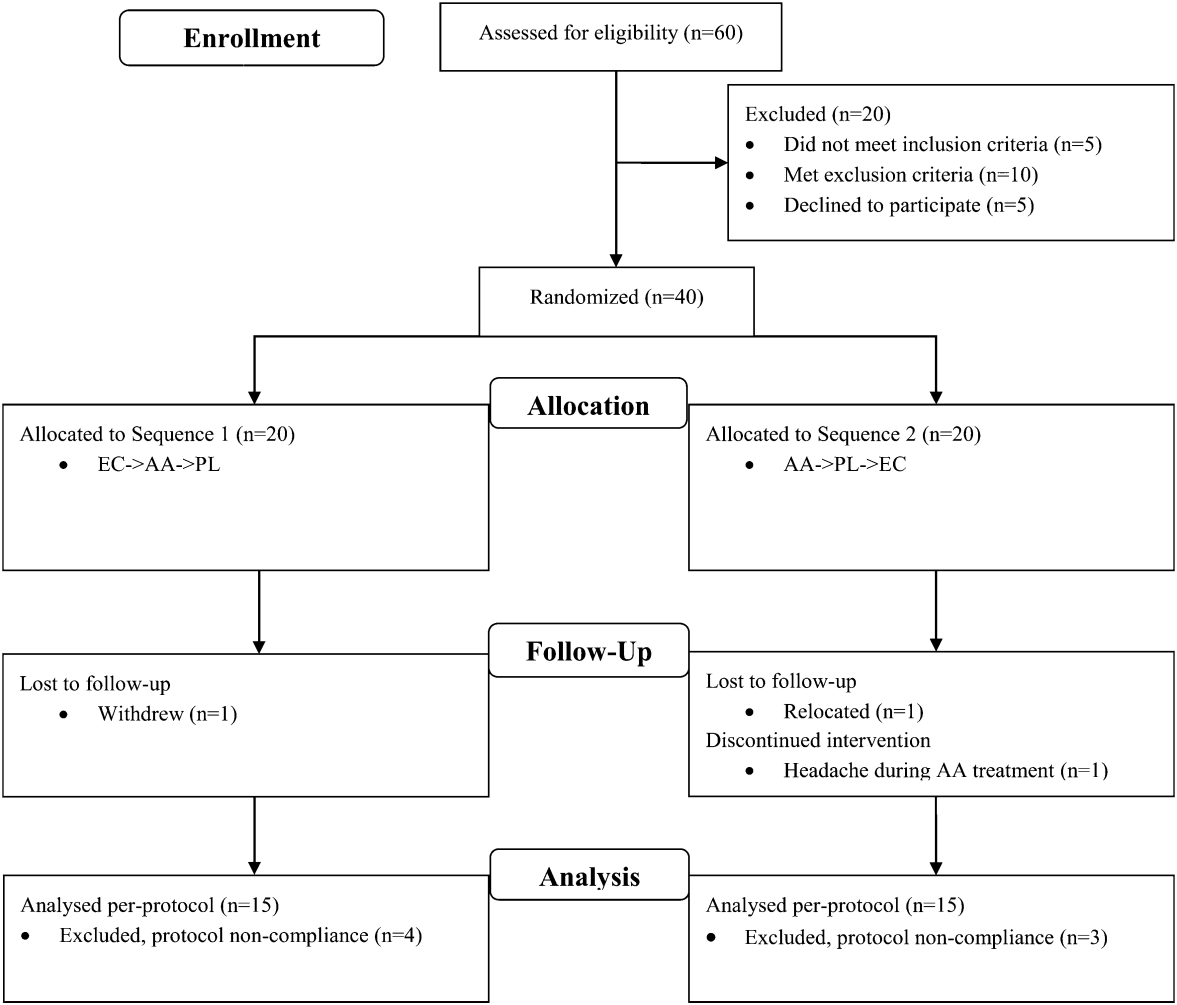
Flow chart of study subjects. *EC* Ester-C^®^, *AA* ascorbic acid, *PL* placebo

Subjects returned to the clinic for randomization and Test Period 1 (Visit 2). The Investigator was provided with two randomization lists (one for male subjects and one for female subjects), indicating the order of randomization to treatment sequence. Each enrolled subject was assigned a randomization code according to the respective randomization list. Each list was prepared using ten blocks of two, with subjects randomized to treatment sequence (Fig. 2) in a 1:1 ratio. The Investigator was provided with sealed envelopes for each randomization code, which was to remain sealed for the duration of the study unless an emergency necessitated unblinding by the investigator. Products were packaged for each individual subject, coded with a randomization number, labeled “Treatment 1, 2 or 3”, and administered at Visits 2, 4, and 6, respectively. All tablets were similar in shape, size and color to ensure adequate blinding of both subject and investigator.

On the first day of each Test Period (Visits 2, 4 and 6), fasting blood samples were collected prior to treatment administration. Immediately after blood collection and just before breakfast, subjects were given two tablets according to their sequence allocation (Fig. 1). Treatments were administered in the presence of clinic personnel to ensure compliance. Subjects remained at the clinic for post-dose blood samples taken at 2, 4, and 8 h. Meanwhile, subjects were provided with standardized low-vitamin C meals (Table 1) for breakfast and lunch during the first 8 h of blood sampling, and the food consumed at each meal was recorded. Subjects were allowed to leave the clinic upon completion of blood sampling. Subjects returned the next day for the 24-h blood collection (Visits 3, 5, and 7). Subjects were counseled and provided with a list of low-vitamin C foods to be consumed while away from the clinic during Test Periods and during the washout periods. Subjects completed food records and submitted them at Visits 3, 5 and 7 to ensure adherence to a low-vitamin C diet. Additionally, each subject was required to record their dietary intake for 2 weekdays and 1 weekend day during each 7-day washout period. Subjects were also advised to refrain from consuming caffeine and alcohol during Test Periods. At the end of the study (after Visit 7), subjects were advised to return to their normal diet.

**Table 1:** Meal choices offered in the clinic on test days (Visits 2, 4 and 6). Adapted from King et al. (1997)

Breakfast	Lunch
Foods	Foods
Cereal (raisin bran, shredded wheat)	Soup (chicken noodle, cream of chicken)
Yogurt (plain, strawberry, blueberry, vanilla)	Wrap (tuna, egg, chicken)
Mini bagel	Yogurt (plain, strawberry, blueberry, vanilla)
Toast (wheat, white)	Bread (wheat, white)
Cream cheese (whole, low-fat)	Saltines
Margarine	Pretzels
Butter	Margarine
Honey	Butter
Peanut butter	Peanut butter
Sugar substitutes	Diet Jell-O
Cream	Chocolate chip cookie
	Ice cream (chocolate, vanilla)
	Sugar substitutes
	Cream
Beverages	Beverages
Decaffeinated coffee	Decaffeinated coffee
Decaffeinated tea	Decaffeinated tea
Milk (whole, 2 %, skim, chocolate)	Ginger ale

### Measurements

Blood samples collected at baseline (0 h) and 2, 4, 8 and 24 h post-dose were analyzed for the pharmacokinetics of plasma and leukocyte vitamin C. Blood was collected in a 6 mL sodium heparin tube (BD Vacutainer, Missisagua, Ontario) and inverted several times to ensure complete mixture of blood and anti-coagulant. The tube was centrifuged at 4 °C for 10 min at 3000 rpm to separate the plasma and buffy coat layers. The following parameters were assessed: area under the curve (AUC_0–24h_), maximum observed concentration (C_max_), time of maximum concentration (T_max_), mean and percent changes of vitamin C from baseline (0 h) in plasma and leukocytes.

#### Plasma vitamin C measurements

A 0.5 mL aliquot of plasma was transferred to a storage vial containing an equal amount of 10 % meta-phosphoric acid with 2 mM ethylenediaminetetraacetic acid (EDTA), covered with foil, frozen at −40 °C, and then stored at ≤−15 °C until analysis. The sample was allowed to thaw at room temperature and 100uL plasma was added to 100 μL of cold 10 % (1:1; v/v) perchloric acid (PCA) containing 1 % mPA (1/v). The mixture was vortexed for 1 min and centrifuged for 5 min at 12,000 RPM. 500 μL of mobile phase was added, the mixture was vortexed and centrifuged again, and 20 μL of the supernatant was introduced into high performance liquid chromatography (HPLC) for analysis of vitamin C with UV absorbance at 245 nm (Rumelin et al. 1999).

#### Leukocyte vitamin C measurements

The buffy coat layer was transferred to 2 mL cryovials and stored at −40 °C until analysis. A 500 µL aliquot was taken and 500 µL of 10 % PCA with 1 % metaphosphoric acid (MPA) was added. The mixture was vortexed in foil for 1 min. 500 µL of mobile phase was added and the mixture was vortexed for another minute and centrifuged for 10 min at 12,000 RPM. 20 µL supernatant was introduced into HPLC for analysis (Emadi-Konjin et al. 2005).

### Statistical analysis

The sample size calculated for this study was based on the article by Moyad et al. (2008). With a conservative standard deviation of 70 and 20 % attrition rate, 40 subjects were determined necessary for 80 % power at the 5 % level of significance.

Baseline comparisons among treatment sequences were analyzed using Chi square test for categorical variables and unpaired t test for continuous variables. AUC_0–24h_, C_max_ and mean concentration in plasma and leukocytes were corrected from baseline and the comparisons were performed on log-transformed data. To minimize substantial variability, outliers in the data set were imputed with the mean value for each time point in each study arm. An outlier was defined as a number that deviated by three standard deviations from the mean. The AUC_0–24h_, C_max_, T_max_, mean and percent changes from baseline were compared among groups using Analysis of Variance (ANOVA) followed by Dunnett’s test to determine differences between EC and other treatment groups. Within-group changes were compared using paired t test. SAS Version 9.1 was used to perform the statistical analysis and statistically significant difference was determined at 0.05. Subjects that were compliant with the protocol were included in the per-protocol (PP) analysis, which is presented and discussed here.

## Results

### Subjects

A total of 60 subjects were screened and 40 eligible subjects were randomized. Of these, 30 were included in the PP analysis (Fig. 2). There were no significant differences in the demographics and baseline characteristics between the two treatment sequences in terms of age, weight, BMI, gender and baseline vitamin C status (Table 2). No premature unblinding occurred during the study.

**Table 2:** Subject demographics and baseline characteristics

Category	EC → AA → PL (N = 15)	AA → PL → EC (N = 15)	P value*
Age (years)	39.87 ± 15.99	43.27 ± 11.92	0.514
Weight (kg)	69.42 ± 11.77	72.76 ± 12.99	0.467
BMI (kg/m^2^)	24.28 ± 3.09	24.71 ± 2.21	0.662
Gender			
Female	7/15 (46.67 %)	7/15 (46.67 %)	0.714^†^
Male	8/15 (53.33 %)	8/15 (53.33 %)	
Baseline vitamin C			
Plasma vitamin C (μg/mL)	6.97 ± 5.42	4.67 ± 3.99	0.197
Leukocyte vitamin C (μg/10^8^ cells)	9.28 ± 6.24	8.73 ± 4.13	0.778

Data are mean ± standard deviation, or frequency (%) for categorical variables

*EC* Ester-C^®^, *AA* ascorbic acid, *PL* placebo

Significant differences are indicated by P values: * means of continuous variables between sequences (compared using an unpaired t test); ^†^ categorical variables between sequences (compared using Chi square test)

### Plasma vitamin C

#### Concentration and percent changes in plasma

**Fig. 3:**
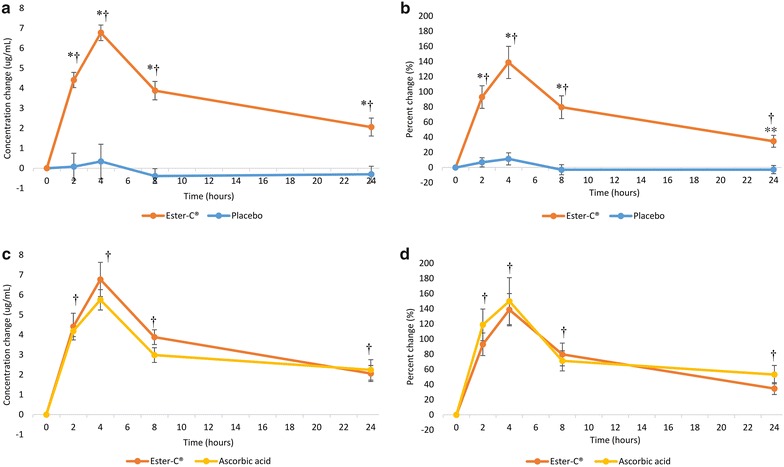
Mean vitamin C concentration and percent changes from baseline in plasma over a 24-h period: **a** concentration change, Ester-C^®^ versus placebo; **b** percent change, Ester-C^®^ versus placebo; **c** concentration change, Ester-C^®^ versus ascorbic acid; **d** percent change, Ester-C^®^ versus ascorbic acid. Data are mean ± standard error. Significant differences are indicated: *P < 0.001, Ester-C^®^ versus placebo; **P = 0.007, Ester-C^®^ versus placebo; ^†^P < 0.001, within-group change from baseline for both Ester-C^®^ and ascorbic acid

The mean concentration and percent changes in plasma from baseline were significantly higher with EC at all time points (P = 0.007 for percent change at 24 h, all others P < 0.001) when compared to the PL condition (Fig. 3a, b). Changes from baseline were similar for EC and AA in plasma, with the peak change occurring at 4 h; and no significant differences between the two groups were observed at any time point (Fig. 3c, d). Compared to baseline, significant increases (P < 0.001) in mean plasma vitamin C concentration and percent change were seen at 2, 4, 8, and 24 h for EC and AA but not for PL (Fig. 3).

#### C_max_, AUC_0–24h_, and T_max_ in plasma

**Fig. 4:**
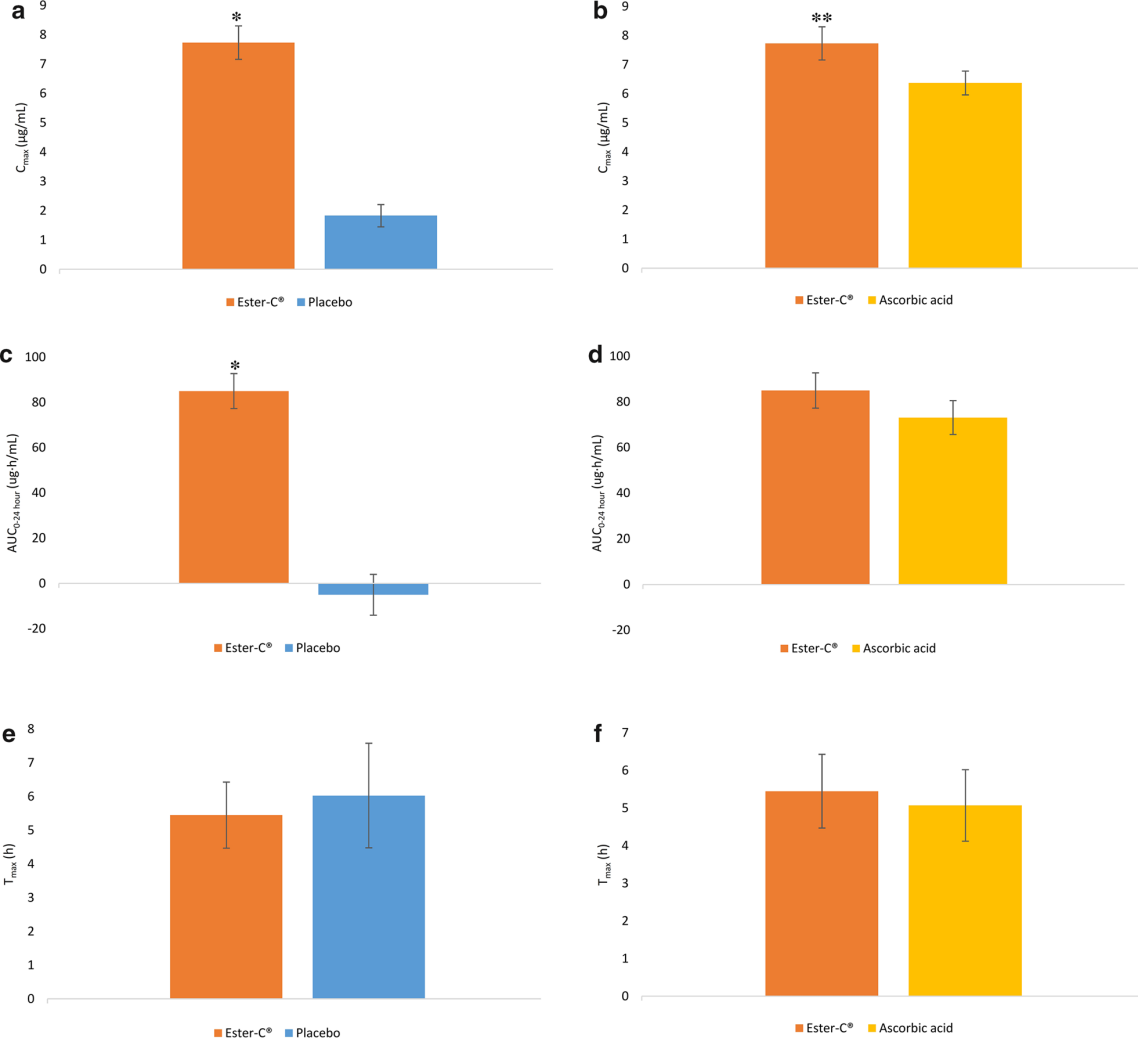
C_max_, AUC_0–24h_, and T_max_ of plasma vitamin C (correcting for baseline) over a 24-h period: **a** C_max_, Ester-C^®^ versus placebo; **b** C_max_, Ester-C^®^ versus ascorbic acid; **c** AUC_0–24h_, Ester-C^®^ versus placebo; **d** AUC_0–24h_, Ester-C^®^ versus ascorbic acid; **e** T_max_, Ester-C^®^ versus placebo; **f** T_max_, Ester-C^®^ versus ascorbic acid. Data are mean ± standard error. Significant differences are indicated: *P < 0.001, Ester-C^®^ versus placebo; **P = 0.039, Ester-C^®^ versus ascorbic acid

C_max_ was significantly higher with EC (7.73 ± 3.12 µg/mL) compared to the PL (1.83 ± 2.07 µg/mL, P < 0.001) and AA (6.37 ± 2.26 µg/mL, P = 0.039) (Fig. 4a, b). The AUC_0–24h_ was significantly higher with EC (85.01 ± 42.39 µg h/mL) compared to PL (−5.01 ± 49.47 µg h/mL, P < 0.001) but not when compared to the AA condition (73.15 ± 40.68 µg h/mL) (Fig. 4c, d). There were no statistical differences in T_max_ between groups (Fig. 4e, f).

### Leukocyte vitamin C

#### Concentration and percent changes in leukocytes

**Fig. 5:**
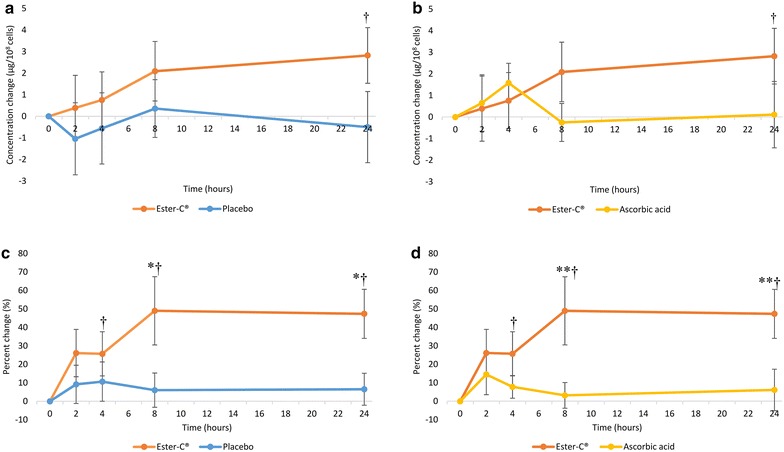
Mean vitamin C concentration and percent changes in leukocytes over a 24-h period: **a** concentration change, Ester-C^®^ versus placebo; **b** concentration change, Ester-C^®^ versus ascorbic acid; **c** percent change, Ester-C^®^ versus placebo; **d** percent change, Ester-C^®^ versus ascorbic acid. Data are mean ± standard error. Significant differences are indicated: *P < 0.05, Ester-C^®^ versus placebo percent change (P = 0.042 at 8 h, P = 0.036 at 24 h); **P < 0.05, Ester-C^®^ versus ascorbic acid percent change (P = 0.028 at 8 h, P = 0.034 at 24 h); ^†^P < 0.05, Ester-C^®^ within-group differences (concentration change P = 0.036 at 24 h; percent change P = 0.040, P = 0.013, and P = 0.001 at 4, 8, and 24 h, respectively)

The mean concentration change from baseline in leukocyte vitamin C was significantly increased with EC at 24 h post-dose (P = 0.036, Fig. 5a, b). No significant within-group changes were observed in PL (Fig. 5a) or AA (Fig. 5b) at any time point. The mean changes in leukocyte vitamin C were numerically higher with EC at 2, 4, 8 and 24 h compared to PL, and at 8 and 24 h compared to AA but did not reach statistical significance (Fig. 5a, b, respectively).

The percent concentration change from baseline in leukocyte vitamin C was significantly higher with EC at 8 and 24 h compared to PL (P = 0.042 and P = 0.036, respectively, Fig. 5c) and AA (P = 0.028 and P = 0.034, respectively, Fig. 5d). The within-group percent change from baseline in leukocyte vitamin C concentration significantly increased with EC at 4, 8, and 24 h post dose (P = 0.040, P = 0.013, and P = 0.001, respectively) (Fig. 5c, d). No significant within-group changes were observed with AA or PL.

#### C_max_, AUC_0–24h_, and T_max_ in leukocytes

The AUC_0–24h_ of the leukocyte vitamin C concentration, though not statistically significant, was highest for EC (47.32 ± 147.24 μg h/10^8^ cells), followed by AA (4.55 ± 102.77 µg h/10^8^ cells) and PL (−4.07 ± 146.86 µg h/10^8^ cells). There were no statistical differences in C_max_ or T_max_ among groups.

### Safety

Adverse events were assessed for all subjects enrolled in the study (n = 40), regardless of whether or not they were compliant with the study protocol. A total of 7 adverse events in 6 subjects were reported (AA, n = 4; EC, n = 2; PL, n = 1). Notably, none of the adverse events were assessed to be related to the investigational products.

## Discussion

This crossover study examined vitamin C bioavailability and retention following a single oral administration of 1000 mg vitamin C provided by EC or AA as measured in plasma and leukocyte vitamin C concentrations. As expected, plasma vitamin C increased significantly from baseline at all time points when subjects were given EC and AA, but not when given PL. There was no significant difference in plasma vitamin C change between EC and AA, but significantly higher plasma C_max_ was observed when subjects consumed EC compared to AA. EC lead to a sustained retention of leukocyte vitamin C during the investigational period, with percent changes from baseline that were significantly higher at 8 and 24 h post-dose compared to AA or PL. In contrast, AA resulted in poor leukocyte vitamin C retention, as indicated by a peak at 4 h postdose with a subsequent decline to baseline concentrations by 8 h. Additionally, the percent change in leukocyte vitamin C from baseline was significant at 4, 8, and 24 h with EC, while there were no significant increases with AA or PL. Area under the leukocyte concentration time curve was highest for EC and was 9 times higher than AA; however, values did not reach statistical significance due to subject variability.

Our study confirms that vitamin C from EC is more bioavailable in leukocytes than AA for male and female nonsmokers. These results are consistent with previous studies (unpublished observations) (Moyad et al. 2008; Wright and Kirk 1990). The most recent clinical trial found increased leukocyte vitamin C concentrations following a daily dose of 1000 mg vitamin C from EC compared to AA in healthy men (Moyad et al. 2008). Another double-blind, crossover trial with the same dose reported similar results (unpublished observations). A third trial found increased vitamin C leukocyte concentration at 8 and 24 h and also after 7–10 days of continuous ingestion of 3000 mg vitamin C as EC compared to AA (Wright and Kirk 1990). All three previous trials were done in men. To our knowledge, the present study is the first clinical trial comparing leukocyte vitamin C concentrations after EC and AA treatment in both male and female subjects. With the inclusion of female subjects, there is concern for a fluctuation in vitamin C concentration correlated with changing hormone levels throughout the menstrual cycle (Michos et al. 2006). To control for this potential variability, the trial was designed so that eumenorrheic women had 28 days between Test Periods, and vitamin C levels could be measured during the same phase of their menstrual cycles. Unexpectedly, only post-menopausal women were randomized, eliminating concerns about cyclic fluctuations.

Our study results also confirm that plasma vitamin C concentrations are tightly regulated; plasma vitamin C concentration with EC and AA had similar patterns, with an abrupt increase during the first 4 h and a peak at 4 h post-dose. Similarly, others have reported no difference in plasma vitamin C concentrations after a single dose of 1000 mg vitamin C given in the form of EC or AA in men (Moyad et al. 2008; Pancorbo et al. 2008). Interestingly, using a three-fold higher dose (3000 mg per day), Wright and Kirk (1990) found a higher serum vitamin C level when EC was consumed compared to AA, while Johnston and Luo (1994) reported a lower plasma vitamin C level when given a much lower EC dose (500 mg vitamin C per day). The latter study had a majority of female subjects under the age of 42 year with no evidence of controlling for vitamin C fluctuations during the menstrual cycle; in addition, subjects were pre-treated for two weeks with 1000 mg of vitamin C per day to saturate body stores before they entered the study, while no other studies used vitamin-C saturated subjects (Johnston and Luo 1994). From the evidence above, a dose–response curve may exist—a mega dose of EC, such as 3000 mg, may result in greater vitamin C concentration in plasma compared to AA, while 1000 mg of EC does not lead to a significant difference. Further studies are needed to clarify this dose–response relationship.

Under physiological conditions, vitamin C exists as both ascorbate (reduced form) and dehydroascorbate (DHA, oxidized form). It has been suggested that vitamin C is predominantly taken up by leukocytes in the form of DHA via a passive, energy-independent, gradient-driven process (Li and Schellhorn 2007). This gradient is influenced by the initial oxidation of vitamin C into DHA and the subsequent reduction into AA after entry into the cell. This allows leukocytes to store vitamin C at higher concentrations than those seen in plasma (Padayatty et al. 2007), which is important for proper cellular function (Bergsten et al. 1990). The vitamin C metabolites found in EC, generated after oxidation to DHA, are thought to further stabilize the extracellular oxidation and metabolism of DHA and/or enhance the passive, facilitated or active transport signaling mechanisms required for intracellular access (Moyad et al. 2008). Past research has indicated that the metabolites in EC help with the transport and utilization of vitamin C (Fay and Verlangieri 1991; Fay et al. 1994; Bush and Verlangieri 1987).

The increased retention of vitamin C in leukocytes is conducive to providing maximum cellular concentrations of vitamin C for optimum biochemical activity, including immune function. In a study with two groups of non-ascorbate synthesizing rats, EC fed animals showed higher vitamin C activity and body weight gain compared to the group fed AA (Verlangieri et al. 1991). In humans, the fundamental processes of leukocytes, especially neutrophil phagocytic capacity, can be depressed when leukocytes are low in vitamin C, however, repletion with vitamin C can help restore these important functions (Jayachandran et al. 2000). Diminished neutrophil function in subjects with furunculosis was improved following supplementation with 1000 mg vitamin C for 4–6 weeks, including increased neutrophil chemotaxis and phagocytosis (Levy et al. 1996). Enhancement of neutrophil motility, chemotaxis and phagocytosis has been observed after the ingestion of 250–3000 mg vitamin C in both healthy and diseased adults (Levy et al. 1996; Anderson et al. 1980). To our knowledge, only one published clinical trial has reported the disease treatment roles of EC—the study showed that compared to placebo, EC (1000 mg of vitamin C per day) can significantly reduce colds and shorten the duration of severe symptoms in winter over a 60-day period (Van Straten and Josling 2002). Further studies are needed to better understand how the level of vitamin C in leukocytes affects neutrophil function and is associated with disease prevention and treatment. The leukocyte vitamin C concentrations reported in our study can serve as future reference values.

While the current study did not measure urinary excretion of vitamin C, previous research is inconsistent. Wright et al. reported less urinary loss with 3000 mg of vitamin C from EC in the first 24 h and after 7–10 days of continuous ingestion when compared to AA (Wright and Kirk 1990). However, these results are not consistent with another study which found similar urinary excretion from EC and AA when only 500 mg of vitamin C was consumed daily (Johnston and Luo 1994). The latter study did not measure vitamin C levels in tissues, so it is possible that more vitamin C from EC moved into tissues (such as leukocytes) and thus yielded similar urinary loss and less plasma vitamin C when compared to AA (Johnston and Luo 1994).

There are a few limitations to our study. There was an observed variability in subjects’ vitamin C levels. Baseline plasma and leukocyte vitamin C concentrations showed a large standard deviation between subjects at study entry (Table 2); however, not significantly different between groups. This was anticipated as these values are dependent on many non-modifiable and modifiable factors (Li and Schellhorn 2007). To address this, measures were taken during this study to minimize the variability by using a crossover design, enrolling only non-smokers, providing meals during test periods and intensely counseling subjects to eliminate dietary vitamin C. The change from baseline and percent change from baseline in plasma and leukocyte vitamin C for each individual subject provided a more accurate assessment of the pharmacokinetics of the treatments. Further, we measured vitamin C levels after a single bolus dose. As shown in a previous trial, vitamin C concentrations in leukocytes were significantly higher at 8 and 24 h when treated with EC and the significant difference continued after 7–10 days of vitamin C consumption at 3000 mg (Wright and Kirk 1990). Future studies should investigate a continuous ingestion of various sources of vitamin C at a lower daily dose.

## Conclusions

In conclusion, EC significantly increased leukocyte vitamin C levels compared to AA and PL. This may be due to the metabolites in EC, which have been shown to facilitate absorption and enhance retention. The superior bioavailability of EC in leukocytes may be beneficial to overall immune function since intracellular vitamin C levels are vital to the fundamental process of leukocytes. More studies are needed to better understand how EC is associated with disease prevention and treatment.

## Abbreviations

AA: ascorbic acid
ANOVA: analysis of variance
AUC: area under the concentration time curve
BMI: body mass index
C_max_: maximum observed concentration
DHA: dehydroascorbic acid/dehydroascorbate
EC: Ester-C^®^
EDTA: ethylenediaminetetraacetic acid
H: hour(s)
HPLC: high performance liquid chromatography
ICH: International Conference on Harmonisation
MPA: metaphosphoric acid
NHPD: Natural Health Products Directorate
NSAIDs: nonsteroidal anti-inflammatory drugs
PCA: perchloric acid
PL: placebo
PP: per-protocol
T: time
T_max_: time of maximum concentration
TP: test period
